# Identification of potential biological targets of oxindole scaffolds via *in silico* repositioning strategies

**DOI:** 10.12688/f1000research.109017.2

**Published:** 2022-03-23

**Authors:** Annachiara Tinivella, Luca Pinzi, Guido Gambacorta, Ian Baxendale, Giulio Rastelli

**Affiliations:** 1 Clinical and Experimental Medicine PhD Program, University of Modena and Reggio Emilia, Modena, Italy; 2 Department of Life Sciences, University of Modena and Reggio Emilia, Modena, Italy; 3 Department of Chemistry, University of Durham, Durham, UK

**Keywords:** drug repurposing, ligand-based, molecular docking, data mining, synthetic products

## Abstract

**Background: **Drug repurposing is an alternative strategy to traditional drug discovery that aims at predicting new uses for already existing drugs or clinical candidates. Drug repurposing has many advantages over traditional drug development, such as reduced attrition rates, time and costs. This is especially the case considering that most drugs investigated for repurposing have already been assessed for their safety in clinical trials. Repurposing campaigns can also be designed for libraries of already synthesized molecules at different levels of biological experimentation, from *null* to *in vitro* and *in vivo*. Such an extension of the “repurposing” concept is expected to provide significant advantages for the identification of novel drugs, as the synthetic accessibility of the desired compounds is often one of the limiting factors in the traditional drug discovery pipeline.

**Methods: **In this work, we performed a computational repurposing campaign on a library of previously synthesized oxindole-based compounds, in order to identify potential new targets for this versatile scaffold. To this aim, ligand-based approaches were firstly applied to evaluate the similarity degree of the investigated compound library, with respect to ligands extracted from the DrugBank, Protein Data Bank (PDB) and ChEMBL databases. In particular, the 2D fingerprint-based and 3D shape-based similarity profiles were evaluated and compared for the oxindole derivates.

**Results: **The analyses predicted a set of potential candidate targets for repurposing, some of them emerging by consensus of different computational analyses. One of the identified targets, i.e., the vascular endothelial growth factor receptor 2 (VEGFR-2) kinase, was further investigated by means of docking calculations, followed by biological testing of one candidate.

**Conclusions: **While the compound did not show potent inhibitory activity towards VEGFR-2, the study highlighted several other possibilities of therapeutically relevant targets that may be worth of consideration for drug repurposing.

## Introduction

Drug repurposing (also known as drug repositioning) is an alternative strategy to traditional drug discovery, that aims at identifying new uses for existing compounds. ^1^ Drug repurposing exploits already available data to explore marketed drugs towards new therapeutic targets and indications, ^2^ thus allowing to overcome some of the issues often encountered in traditional ( *de novo*) drug discovery. According to a recent report by the United States of America (US) Food and Drug Administration (FDA), the number of approved drugs has been declining since 1995, while investment in drug development continues to increase. ^3^ Indeed, traditional drug discovery and development processes usually require up to 10 – 15 years and $2 – 3 billions to bring a new drug to the market from its first identification. However, the success rate of developing a new drug is only around 2%, ^3^ with the main causes behind the end of candidate research programs being safety or efficacy issues. ^3^ Moreover, approved drugs can also be withdrawn due to side effects discovered by post-market monitoring. On the other hand, drug repurposing usually requires only preclinical and clinical testing to place drugs again on the market, according to literature data, ^1^ as it focuses on molecules that have already passed preclinical and clinical experimentations. Hence, drug repurposing is less expensive with respect to traditional drug discovery. This is especially true when considering that safety and formulation assessment data are generally available for already marketed drugs. Based on these premises, drug repurposing can be considered a potentially faster and cost-effective approach, with lower risks of failure compared to traditional *de novo* design. ^1^^,^^4^

The application of drug repurposing strategies can also be extended to clinical candidates, natural products, and already synthesized compounds. ^3^^,^^4^ Compounds whose scaffolds have already reported synthetic accessibility represent a valuable source for repositioning towards novel targets and therapeutic applications, especially considering that their preparation often represents a rate-limiting factor in several *de novo* drug discovery projects. ^5^ Indeed, the identification of novel biological targets for already synthesized chemical scaffolds may allow to maximize their value for drug discovery and to help circumvent potential issues often encountered when synthesizing novel compounds. In recent years, computational approaches have proved to be valuable tools for the development of repositioning campaigns, especially when applied in integrated workflows. ^6^^–^^8^ The possibilities offered by *in silico* repositioning approaches for the fast identification of novel potential therapeutic remedies have also come into play in the current COVID-19 pandemics. ^9^^–^^12^ In particular, a vast number of approaches have been applied to SARS-CoV-2-related targets ^9^^–^^11^ and to analyze clinically relevant information in search of effective treatments against COVID-19. ^13^^,^^14^ Moreover, novel valuable therapeutic opportunities have also emerged from the application of computational repositioning strategies to anticancer and anti-infective drug discovery. ^7^^,^^15^^–^^17^

Based on these premises, in this work we set up and applied a repurposing campaign with the aim of identifying suitable targets for a library of previously synthesized oxindole derivates. ^18^ The oxindole ring represents a privileged scaffold in nature, being ubiquitously present in tissues and in mammalian body fluids, as well as in plant-derived natural products, bacteria and invertebrates. ^19^ A repurposing workflow integrating different computational approaches was implemented in order to increase the robustness of the adopted *in silico* protocol, ^6^ such a procedure having already demonstrated to increase the screening performances in polypharmacology contexts. ^20^ In particular, we firstly carried out extensive 2D fingerprint- and 3D shape-based similarity estimations on the investigated library of compounds against ligands with a known profile of activity, extracted from the DrugBank, Protein Data Bank and ChEMBL databases. ^21^^–^^23^ Interestingly, several targets emerged as valuable candidates for the repositioning of the compounds. Then, we further investigated one of the identified targets by means of structure-based approaches such as rigid and induced-fit docking. Besides, we also performed quantum-mechanical analyses on the ligands to identify their more favourable tautomer at physiological pH, to be used in the docking calculations. Finally, one candidate was experimentally tested on the identified target.

## Methods

### Ligand-based calculations

The library consisting of the oxindole-based compounds and their tautomers was designed by using Maestro of the Schrödinger Suite. For an open-source software alternative, users can utilize for example AutoDockTools from the AutoDock suite. The DrugBank, Protein Data Bank ( PDB) and ChEMBL ligands were first downloaded from their respective databases (accessed on October 13 ^th^, 2020) and filtered as follows. ^21^^–^^23^ With regards to the DrugBank entries, only small molecules were retained during the filtering process and considered for the ligand-based analyses. The PDB ligand dataset was processed to remove crystallization adjuvants, solvent molecules, and ions. Finally, the ChEMBL dataset was filtered to retain only molecules with data deriving from experiments conducted in the “Single Protein” format, and bioactivity expressed as K _i_, IC _50_, K _d_, EC _50_ or Potency. The molecules filtered from ChEMBL were also classified as active or inactive, according to an activity threshold equal to 1,000 nM. Moreover, duplicates records reported for the ChEMBL molecules were removed, retaining only the most frequent activity label of each ligand.

Afterwards, the prepared ligand datasets were subjected to a series of ligand-based similarity estimations. In particular, 2D fingerprint-based similarity calculations were performed for the investigated oxindole derivates, against the ligands filtered from DrugBank, PDB and ChEMBL, by using an *in-house* developed python script relying on the RDKit toolkits. ^24^ The degree of similarity of the compounds was evaluated according to the MACCS, ECFP4, ECFP6, FCFP4 and FCFP6 types of fingerprints, in terms of the Tanimoto coefficient (Tc, ranging from 0 to 1), similarly to that performed in our previous studies ( *e.g.*, see reference 25).

Besides, 3D similarities between the oxindole derivates and the ligands previously filtered from DrugBank, PDB and ChEMBL were also calculated using OMEGA (version 3.1.2.2) ^26^^,^^27^ and ROCS (Rapid Overlay of Chemical Structures, version 3.3.2.2) softwares, ^28^ which are part of the OpenEye suite. For an open-source alternative of a shape-based similarity software, users can utilize for example MolShaCS. The comparisons carried out between the oxindole-compounds and the ligands filtered from the DrugBank and ChEMBL databases were performed as follows. First, up to five conformers were generated for the oxindole compounds with OMEGA, which were considered as the query molecules in these rounds of 3D similarity estimations. On the other hand, a maximum of 50 (defaults settings) and 600 conformers per ligand were generated for the filtered ChEMBL and DrugBank datasets, respectively. A higher number of conformers was generated for the DrugBank ligands, which enabled to perform a broader conformational sampling, thus increasing the possibility to take into consideration the bioactive conformation of the molecules at an affordable calculation time. Defaults parameters were used during the conformational sampling with OMEGA, except for the number of conformers to be generated *per* ligand (see above). Then, 3D similarity calculations were conducted with ROCS using defaults settings. On the contrary, the 3D comparisons between the oxindole derivates and the ligands filtered from PDB were performed by considering these latter compounds in their crystallographic conformations as queries. In this case, up to 50 conformers generated for each oxindole derivate and the 3D similarity estimations were performed by using ROCS with defaults parameters.

### Tautomeric stability

The tautomeric preference of the oxindole compounds was evaluated by using Jaguar, which is a program available in the Schrödinger suite, specialized in fast *ab initio* quantum chemical predictions in gas and solution phases, for molecular systems of medium and large size. ^29^ For an open-source alternative, users can utilize the QMCPACK package. In this work, a structural optimization of the molecules was first performed with default settings. Then, Single Point Energy (SPE) calculations were run to evaluate the energy of structures at their minimum energy geometry. Jaguar calculations were performed using the 6_31 G** basis set and *Density-functional theory (DFT)* with B3LYP-D3 functional, automatic SCF spin treatment, and grids with a medium point density. The *Poisson-Boltzmann Solvation Model* was set as the solvation model, with water as solvent. Other calculation parameters were set to the default.

### Docking into the vascular endothelial growth factor receptor 2 (VEGFR-2)

The 3VID PDB structure of vascular endothelial growth factor receptor 2 (VEGFR-2) was firstly downloaded from the RCSB PDB website and prepared with the *Protein Preparation Wizard* tool available in Maestro, from the Schrödinger suite 2020-2. ^30^ The tool was used to assign bond order, add missing hydrogens and cap termini, and to generate the most suitable protonation and tautomerization states of the protein residues. The optimization of the hydrogen bond networks in the receptor was performed by using PROPKA with physiological pH settings. Water residues beyond 3 Å from the crystallographic ligand were removed, and a final step of restrained minimization was carried out by using the OPLS3e force field, with a convergence of heavy atoms equal to a root-mean-square deviation (RMSD) of 0.3 Å. Then, a grid to be used for molecular docking with Glide (Schrödinger suite 2020-2) was generated at the centroid of the crystallographic ligand. ^31^ The crystallographic ligand ( *i.e.*, PDB HET ID: 4TT) was extracted from the complex and prepared with *LigPrep* along with oxindole_9 and oxindole_19, in order to generate minimized structures with suitable protonation states at a pH of 7±1. ^30^ Molecular docking calculations were then run with Glide, by using the Standard Precision (SP) protocol. ^31^ For an open-source alternative, users can utilize the AutoDock4 software from the AutoDock suite. The number of poses to be subjected to post-docking minimization was increased to 10, while all other parameters were set to the default. Moreover, additional calculations were also performed according to the Induced Fit Docking (IFD) protocol implemented in the Schrödinger suite. ^32^ The IFD procedure allows to account for side chain flexibility in the active site of the receptor. Calculations were run with default settings, leading to up to 20 poses for each compound. For both rigid and semi-flexible docking, the *Gscore* metric was evaluated, and all poses were visually inspected.

### Biological testing on VEGFR-2

Evaluation of *in vitro* activity on VEGFR-2 was performed at Reaction Biology Corp. *via* a radiometric HotSpot™ kinase assay. ^33^ A reaction buffer containing 20 mM 4-(2-hydroxyethyl)-1-piperazineethanesulfonic acid (Hepes, pH 7.5), 10 mM MgCl _2_, 1 mM ethylene glycol-bis(β-aminoethyl ether)-N,N,N′,N′-tetraacetic acid (EGTA), 0.01% Brij35, 0.02 mg/ml bovine serum albumin (BSA), 0.1 mM Na _3_VO _4_, 2 mM dithiothreitol (DTT), and 1% dimethyl sulfoxide (DMSO) was freshly prepared. Then, a 0.2 mg/ml solution of peptide substrate, poly [Glu:Tyr] (4:1) solution was added. After 20 minutes, adenosine triphosphate (ATP, supplied from Sigma, St. Louis, MO, USA) and radioisotope-labeled ATP ( ^33^P-ATP, supplied from PerkinElmer, Waltham, MA, USA) were added to reach a final concentration of 10 μM. Following an incubation time of 2 hours at 25 °C, spotting of the reactions on P81 ion exchange filter paper (supplied from Whatman Inc., Piscataway, NJ, USA) was performed. Excess unbound phosphate was removed, and kinase activity was detected and expressed as the proportion of kinase activity remaining in the test samples relative to the vehicle (DMSO) reactions. IC _50_ values and curve fits were calculated by employing Prism ( GraphPad Software, version 9).

## Results

In this work, we performed a repositioning campaign for a library of ligands based on the oxindole scaffold that we obtained through organic synthesis from a previous study. ^18^ The library was formed by two compounds constituting a racemic pair, in which the oxindole ring is linked to a pyrazole *via* a methylene linker. As both the oxindole and pyrazole rings can exist in different tautomeric states, ^34^ all possible tautomers of the ligands were calculated, obtaining a total of 20 structural items ( Table 1). As it is, the library can be divided in two main enantiomers (enantiomer 1 and enantiomer 2) and all possible tautomers for each enantiomeric species. Table 1 reports the chemical structures of each tautomer and their corresponding ID number.

**Table 1.** Chemical structure of the oxindole derivates and their investigated tautomeric forms.

Pyrazole group	Enantiomer 1		Enantiomer 2	
	Hydroxy indole	Oxindole	Hydroxy indole	Oxindole
N-NH=O	oxindole_4 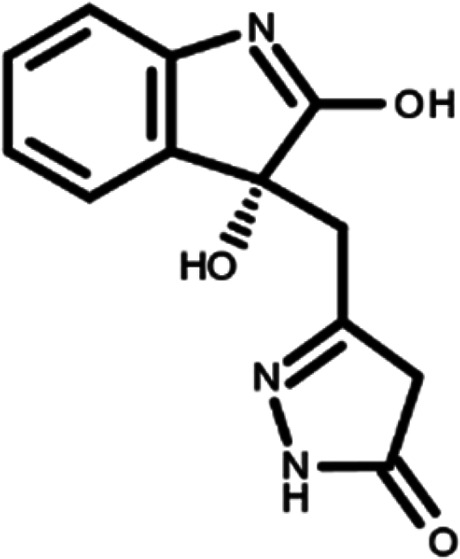	oxindole_9 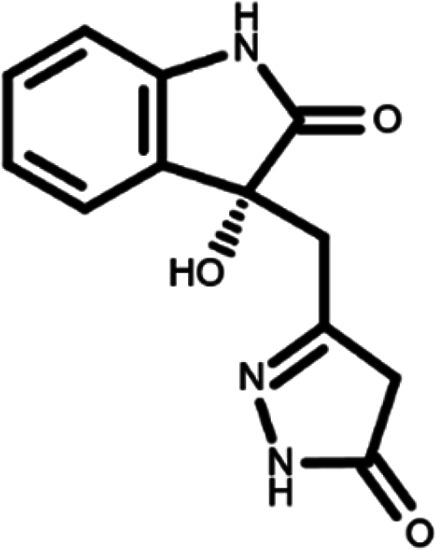	oxindole_14 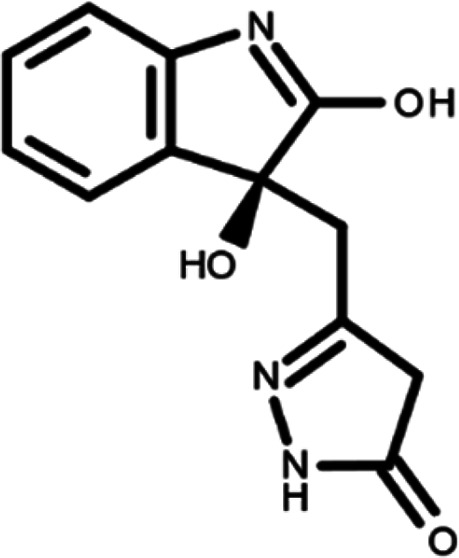	oxindole_19 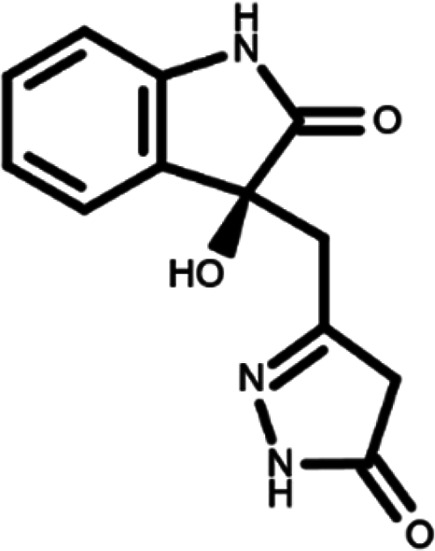
NH-NH=O	oxindole_5 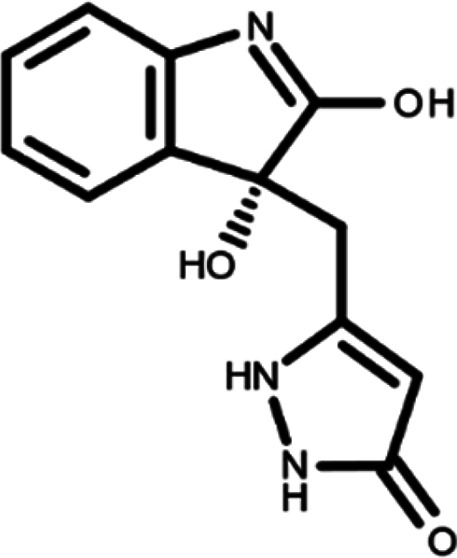	oxindole_10 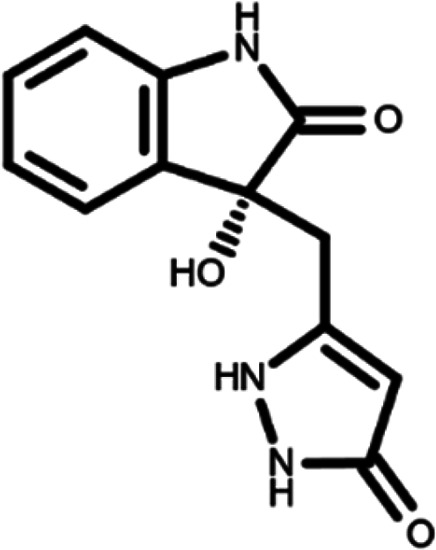	oxindole_15 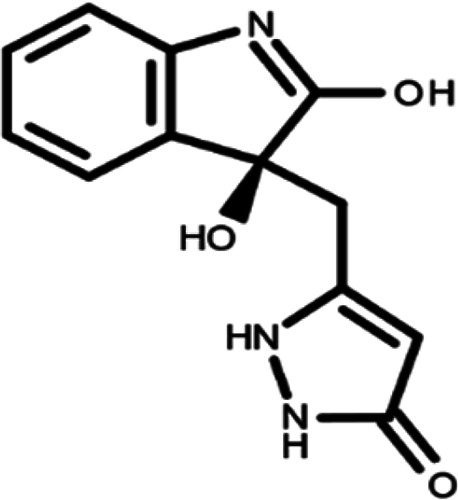	oxindole_20 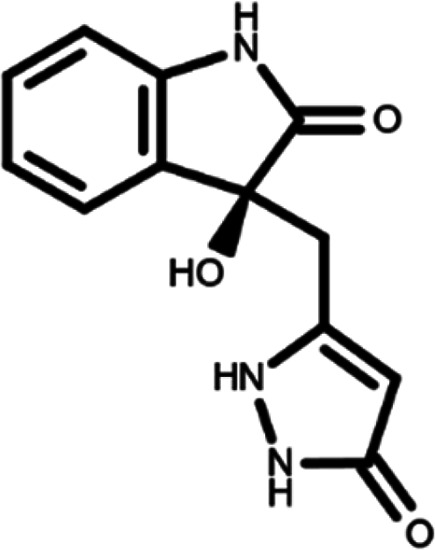
N-NH-OH	oxindole_3 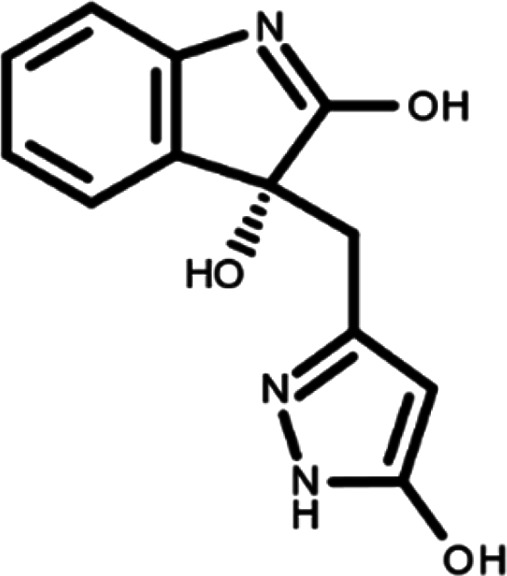	oxindole_8 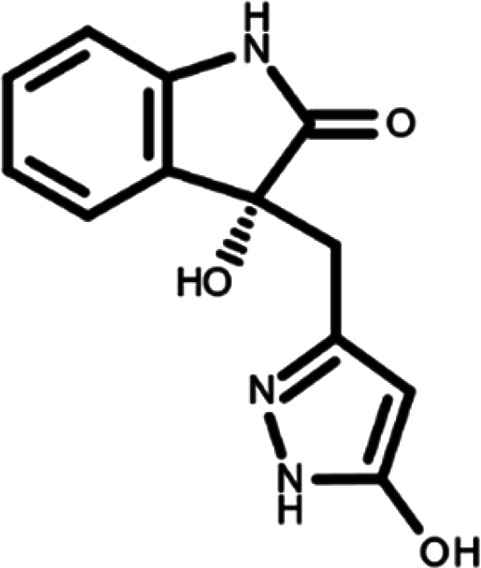	oxindole_13 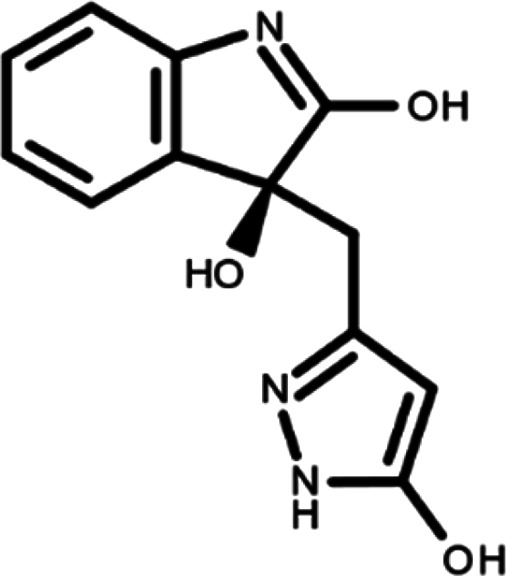	oxindole_18 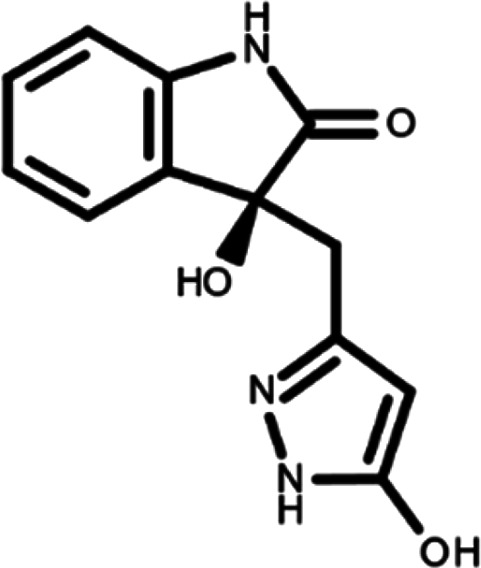
N-N-OH	oxindole_2 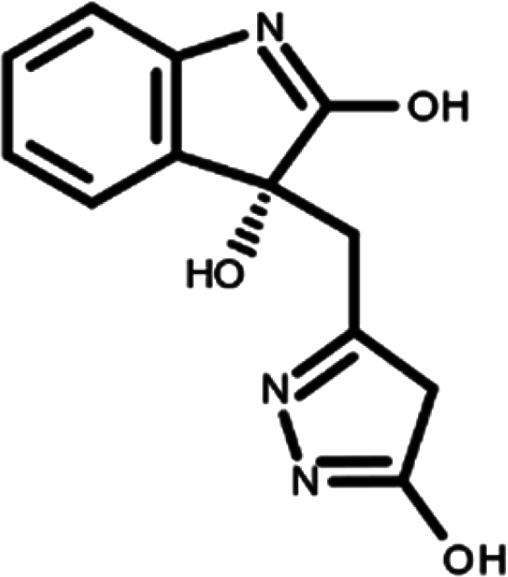	oxindole_7 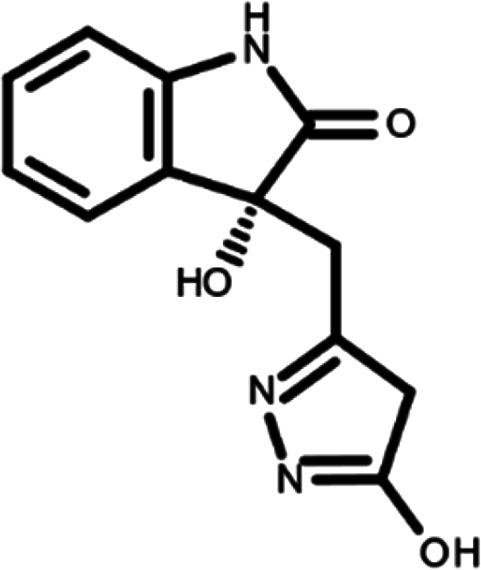	oxindole_12 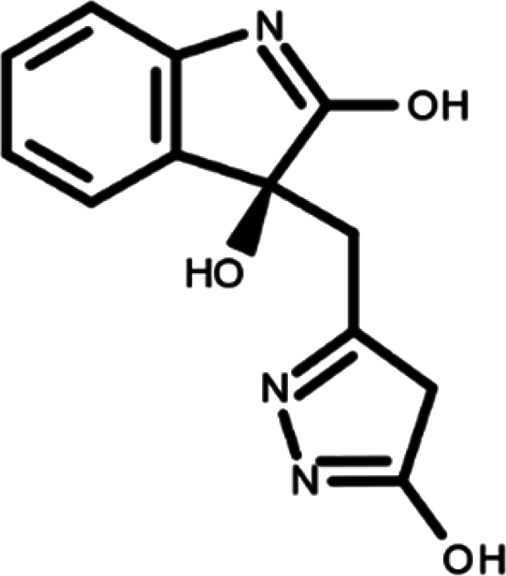	oxindole_17 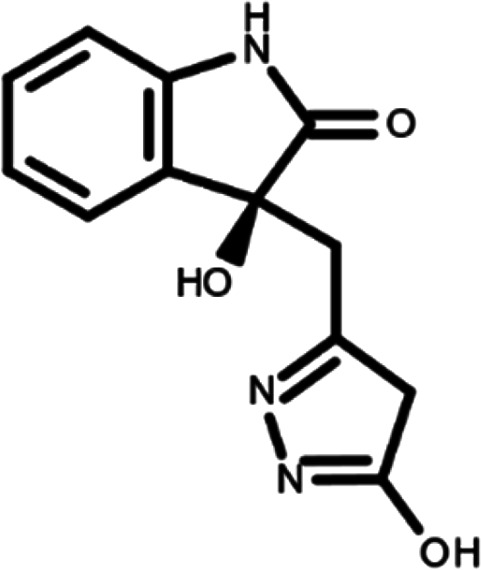
NH-N-OH	oxindole_1 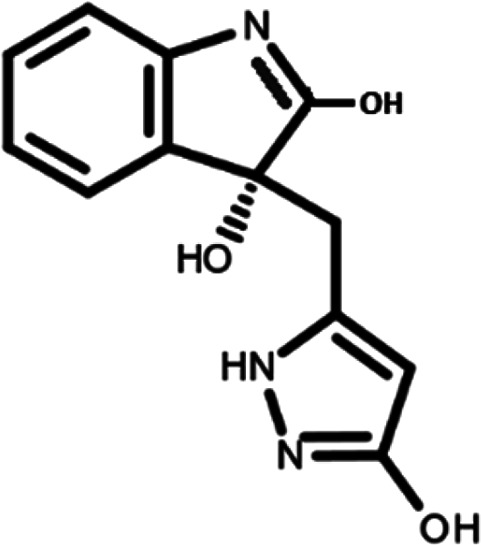	oxindole_6 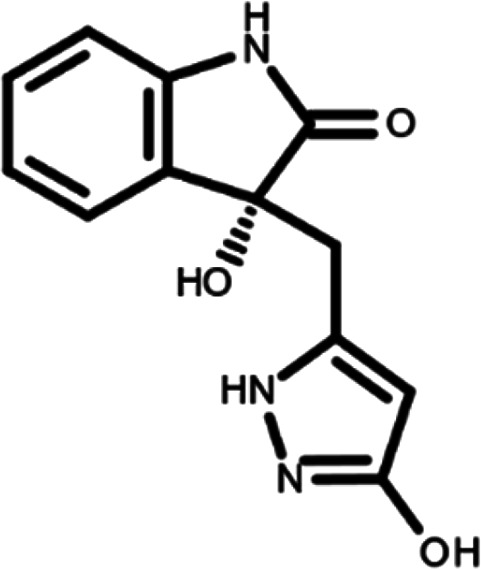	oxindole_11 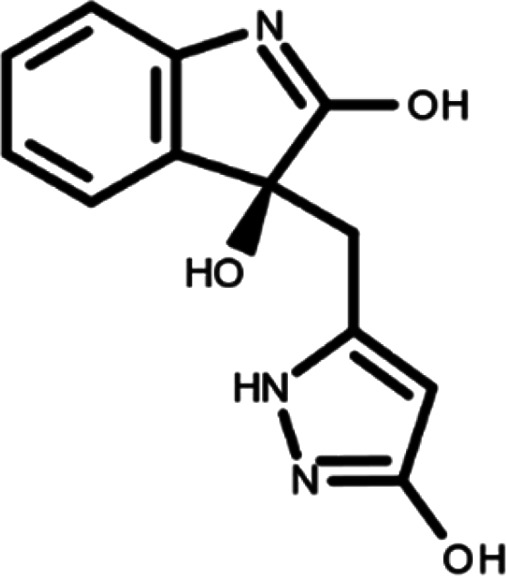	oxindole_16 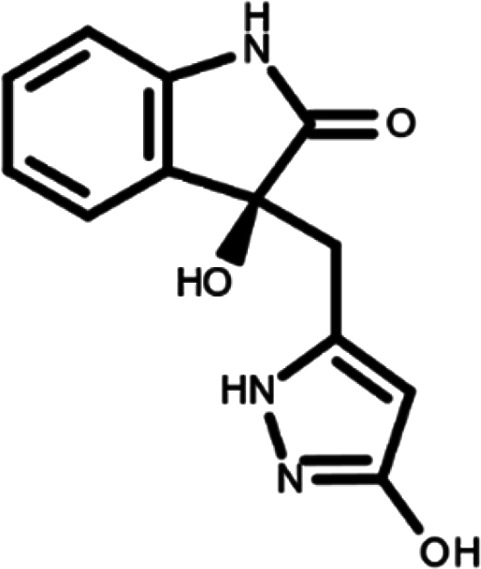

### Ligand similarity analysis

**Figure 1.**
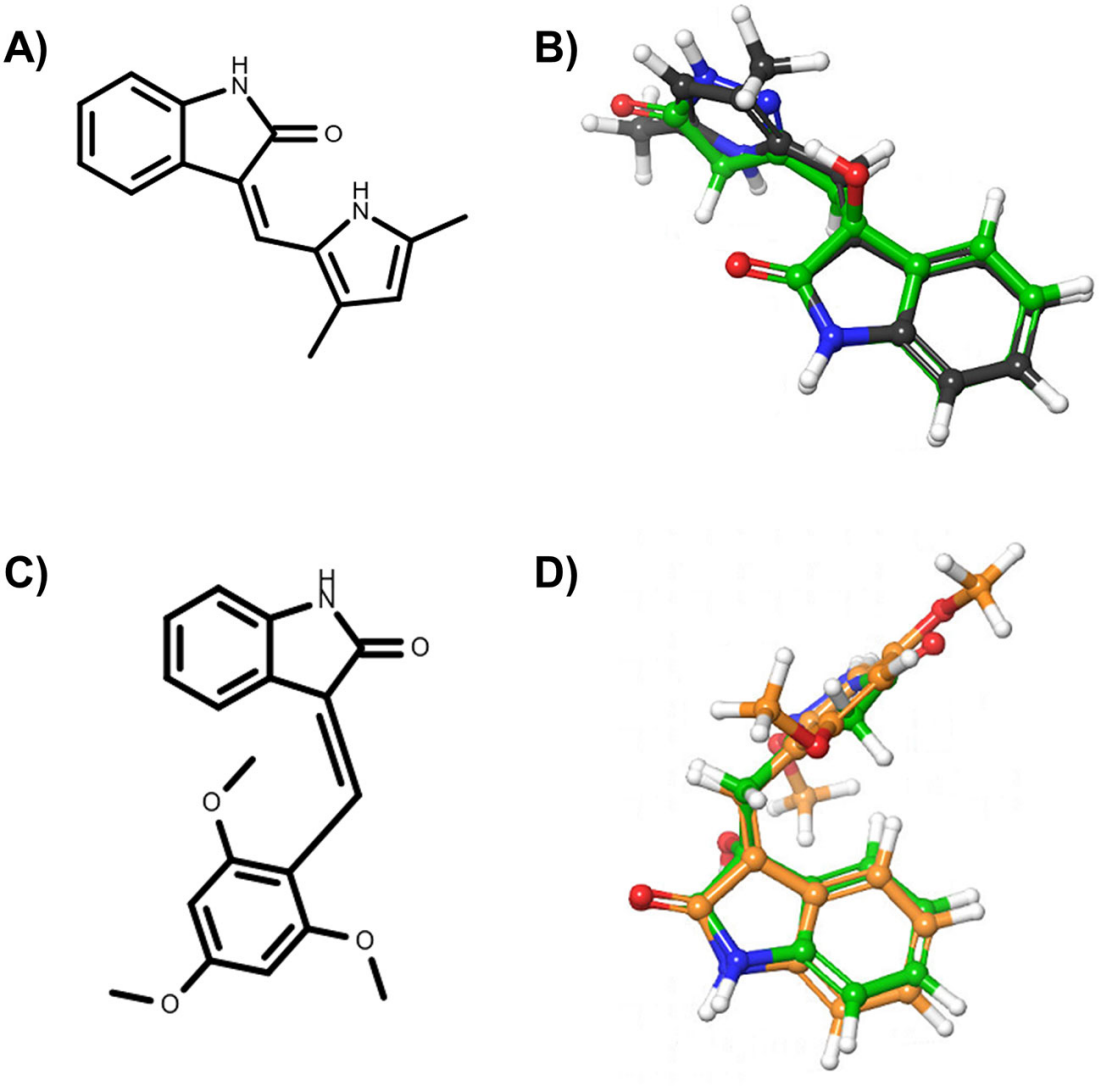
Ligands selected from the consensus of the 2D and 3D similarity analyses. In particular, panel A) reports the chemical structure of semaxinib, while panel B) the superimposition of semaxinib (depicted as grey sticks) on oxindole_19 (depicted as green sticks). Panel C) reports the chemical structure of IC261, while panel D) the superimposition of IC261 (depicted as orange sticks) on oxindole_19 (depicted as green sticks).

In the first part of the *in silico* repurposing screening, we performed a series of 2D fingerprint-based and 3D shape-based ligand-based analyses, to assess the similarity profile of the oxindole molecules with respect to ligands extracted from DrugBank, PDB and ChEMBL. ^21^^–^^23^ In particular, we firstly performed 2D MACCS, ECFP and FCFP fingerprint-based similarity estimations by using an *in-house* python script relying on RDKit toolkits. ^24^ We performed 2D estimations according to different types of fingerprints to better assess the degree of structural similarity between the molecules. Then, the obtained similarity records were filtered to retain only those whose values were above that of activity-relevant Tanimoto coefficient (Tc) similarity thresholds, *i.e.*, Tc _MACCS_ ≥ 0.7, Tc _ECFP4_ ≥ 0.3, Tc _ECFP6_ ≥ 0.4, Tc _FCFP4_ ≥ 0.3 and Tc _FCFP6_ ≥ 0.4. ^35^ The results of 2D similarity analyses related to the best candidates are available as *Extended data.* ^36^ Besides, we carried out 3D similarity calculations by means of the ROCS software as described in the Methods section, retaining only records that provided a TanimotoCombo (TC) coefficient equal or higher than 1.2. Afterwards, the obtained similarity records were merged with KNIME (version 4.2.1), ^37^ retaining only the pairs of molecules ( *i.e.*, oxindoles *versus* DrugBank ligands; oxindoles *versus* PDB ligands; oxindoles *versus* ChEMBL ligands) that provided values above threshold for all types of Tanimoto coefficients. The RDKit ^24^ node available in KNIME was firstly used to convert all molecules in the filtered similarity results into their canonical SMILES strings. Then, these structural annotations were used as keys for joining the results. This step was required to allow the perfect joining of molecules present across the DrugBank, PDB and ChEMBL databases. Interestingly, this analysis allowed to highlight two molecules reported in the DrugBank, PDB and ChEMBL databases, which provided 2D and 3D similarity values above threshold to selected oxindole derivates. In particular, semaxanib ( *i.e.*, DrugBank ID: DB06436, PDB Het ID: X2M and ChEMBL ID: CHEMBL276711) and IC261 ( *i.e.*, DrugBank ID: DB03083, PDB Het ID: IC1 and ChEMBL ID: CHEMBL489156) were found to be similar to the oxindole_7, oxindole_8, oxindole_9, oxindole_10, oxindole_17, oxindole_18, oxindole_19, oxindole_20 derivates, with TanimotoCombo coefficients ranging from 1.3 to 1.5. The structures of semaxanib and IC261 and the superimposition with the oxindole scaffold are shown in Figure 1. Interestingly, semaxanib ( Figure 1A-B) was first identified as an inhibitor of VEGF- and PDGF-induced tyrosine autophosphorylation in a high-throughput screening, and was then confirmed as a VEFGR-2 blocker. ^38^ VEGFR-2 (UniProt ID: P35968) is a tyrosine-protein kinase that plays an essential role in the regulation of angiogenesis, vascular development, and vascular permeability. ^39^ Of note, semaxanib has been reported in crystallographic complex with the proto-oncogene tyrosine-protein kinase receptor Ret (UniProt ID: P07949). Although semaxanib initially represented a promising candidate for the treatment of colorectal cancer, clinical investigations on this compound were lately discontinued, mainly due the severe toxicities observed in phase II/III. ^38^ Besides, IC261 ( Figure 1C-D) is an inhibitor of the casein kinase I isoform γ-2 (UniProt ID: P78368), a receptor tyrosine-protein kinase involved in brain development. ^40^ An analysis of the obtained 2D and 3D similarity records and their comparison with activity data reported for the ligands into the three databases were also performed. This allowed to first identify the targets with the highest number of ligands (either active, or inactive) that were found to be similar to the investigated oxindole ligands. Then, for each target we evaluated the difference between the number of associated molecules reported as active and inactive, prioritizing those with the larger number of more potent inhibitors. In this phase, particular attention was devoted to targets that emerged from the comparison of ligands reported in multiple databases (see Table 2). In particular, good values of similarity were observed with potent inhibitors of VEGFR-2 ( *e.g.*, ChEMBL IDs: CHEMBL276711, CHEMBL101797, CHEMBL328029, CHEMBL193094, CHEMBL191437, CHEMBL393636, CHEMBL426078, CHEMBL408565, CHEMBL89363, CHEMBL147761, CHEMBL344319). Based on these results, we selected VEGFR-2 as a candidate target on which to perform additional *in silico* structure-based analyses.

**Table 2.** Most promising targets identified through the 2D and 3D ligand-based similarity analyses. An activity threshold equal to 1,000 nM was used to discriminate active from inactive ChEMBL ligands.

Uniprot ID	Target name	Target organism	PDB count	DB count	ChEMBL active count	ChEMBL inactive count
**P35968**	**Vascular endothelial growth factor receptor 2**	***Homo sapiens***	**0**	**10**	**11**	**7**
P35354	Prostaglandin G/H synthase 2	*Homo sapiens*	0	10	1	0
P11309	Serine/threonine-protein kinase pim-1	*Homo sapiens*	1	12	0	0
P0A0K8	DNA gyrase subunit B	*Staphylococcus aureus*	1	0	0	1
P56817	β-secretase 1	*Homo sapiens*	1	0	0	1
P11362	Fibroblast growth factor receptor 1 (FGFR-1)	*Homo sapiens*	0	4	1	5
P08684	Cytochrome P450 3A4	*Homo sapiens*	0	2	0	5
P07949	Proto-oncogene tyrosine-protein kinase receptor Ret	*Homo sapiens*	1	0	4	10
P30536	Translocator protein (Mitochondrial benzodiazepine receptor) (PKBS)	*Homo sapiens*	0	2	0	24

### Tautomeric stabilities

It has been reported that the most active tautomer of known tyrosine kinase inhibitors bearing an oxindole scaffold is the ketonic form. ^34^ Moreover, we have previously shown that oxindoles in the ketonic forms oxindole_7, oxindole_8, oxindole_9, oxindole_10, and the corresponding enantiomers oxindole_17, oxindole_18, oxindole_19, oxindole_20 resulted as the most similar to the top-scoring VEGFR-2 inhibitor semaxinib and to 10 other VEGFR-2 potent inhibitors (see above). However, the oxindole compounds could potentially exist in different tautomeric forms at physiological pH. Therefore, we computed the energy in the gas and water solution phases of the investigated library of oxindoles in order to establish which was the most stable tautomer at physiological pH. To this aim, the oxindole derivates were firstly minimized. Then, tautomeric preference in gas and water solutions were computed by using the SPE tool available in Jaguar (Schrödinger), as described in the Methods section. The obtained results ( Table 3) highlighted that the library molecules with the ketonic form of both indole and pyrazole rings ( *i.e.*, oxindole_9 and oxindole_19, respectively) were more stable. For both enantiomers the second more stable tautomer was the one bearing only the oxindole in the ketonic form ( *i.e.*, oxindole_6 and oxindole_16).

**Table 3.** Results of quantum-mechanical Single Point Energy calculations performed for oxindole molecules.

	Gas phase energy (kcal/mol)	Solution phase energy (kcal/mol)	Δ Gas phase energy to most stable oxindole (kcal/mol)	Δ Solution phase energy to most stable oxindole (kcal/mol)
**Enantiomer 1**				
**oxindole_9**	-535798.9	-535825.4	0	0
oxindole_10	-535789.8	-535820.8	9,03	4,57
oxindole_8	-535786.0	-535817.8	12,84	7,63
oxindole_7	-535778.2	-535807.9	20,66	17,52
oxindole_6	-535794.2	-535821.7	4,68	3,70
oxindole_4	-535781.6	-535807.8	17,23	17,64
oxindole_5	-535775.4	-535805.0	23,41	20,36
oxindole_3	-535773.8	-535802.0	25,08	23,37
oxindole_2	-535754.9	-535787.1	43,92	38,29
oxindole_1	-535776.3	-535804.7	22,54	20,68
**Enantiomer 2**				
**oxindole_19**	-535798.0	-535825.7	0	0
oxindole_20	-535790.3	-535821.5	7.7	4.2
oxindole_18	-535786.8	-535817.2	11.2	8.5
oxindole_17	-535776.3	-535807.0	21.7	18.7
oxindole_16	-535792.8	-535820.3	5.2	5.4
oxindole_14	-535783.2	-535808.5	14.9	17.3
oxindole_15	-535770.1	-535799.2	27.9	26.6
oxindole_13	-535773.5	-535800.9	24.5	24.8
oxindole_12	-535757.1	-535789.5	41.0	36.2
oxindole_11	-535778.5	-535804.2	19.5	21.5

### Analysis of vascular endothelial growth factor receptor 2 (VEGFR-2) with structure-based approaches

**Figure 2.**
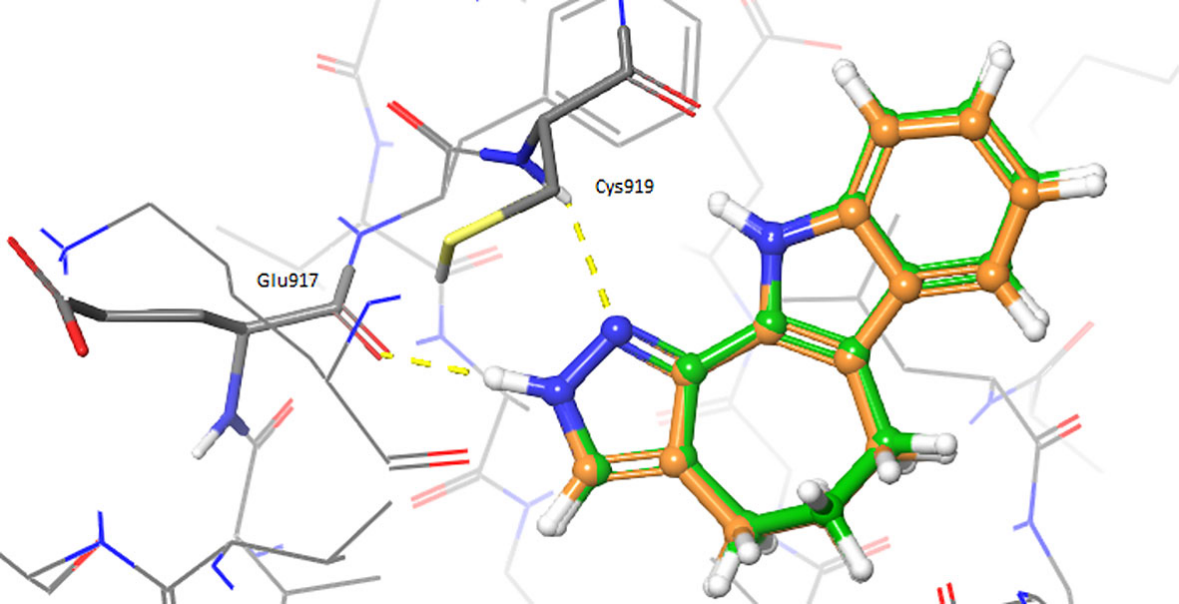
Superimposition of the crystallographic pose of 4TT (depicted as orange sticks) into the vascular endothelial growth factor receptor 2 (VEGFR-2) active site (Protein Data Bank code: 3VID), with the binding mode predicted by docking for the same ligand (depicted as green sticks). Hydrogen bonds are depicted as yellow dashed lines.

The ligand-based analyses allowed to identify VEGFR-2 as a potential target for the repurposing of the oxindole derivates. To evaluate whether the oxindole derivates could also present good electrostatic complementarity with the binding site of VEGFR-2, we performed molecular docking calculations on the crystallographic structure of this target, which has been recently reported in complex with inhibitor 4TT (PDB accession code: 3VID). The 3VID complex was selected among those available for VEGFR-2 as its co-crystallized ligand 4TT resulted to be the most similar to the enantiomeric pair consisting of oxindole_9 and oxindole_19, the TanimotoCombo coefficient observed for these compounds being 1.1. The 3VID crystal structure was first processed as described in the Methods section to generate grids suitable for the rigid and IFD docking calculations. Then, re-docking calculations of 4TT into its parent receptor grid were performed to verify whether the docking software was able to reproduce the experimental binding mode of the crystallographic ligand. The resulting RMSD values calculated between the crystallographic conformation of 4TT and the predicted docking poses into 3VID were below 2.0 Å. Moreover, as can also be observed in Figure 2, the pose predicted for 4TT by re-docking retained all the interactions observed in the 3VID ligand-protein crystallographic complex, thus further validating the generated structure-based models. According to the crystallographic and docking poses, the 4TT ligand formed several hydrophobic interactions with the Leu840, Val848, Ala866, Val916, Phe918, Cys919, Lys920, Gly922 and Leu1035 residues. Moreover, the ligand also established a hydrogen bond with the Cys919 residue and a π-π stacking interaction with Phe918. In particular, the former H-bond interaction with Cys919 is especially important for the activity on VEGFR-2 according to crystallographic and activity data, and as can also be observed for the majority of protein tyrosine kinase (PTK) inhibitors reported in the literature.

**Figure 3.**
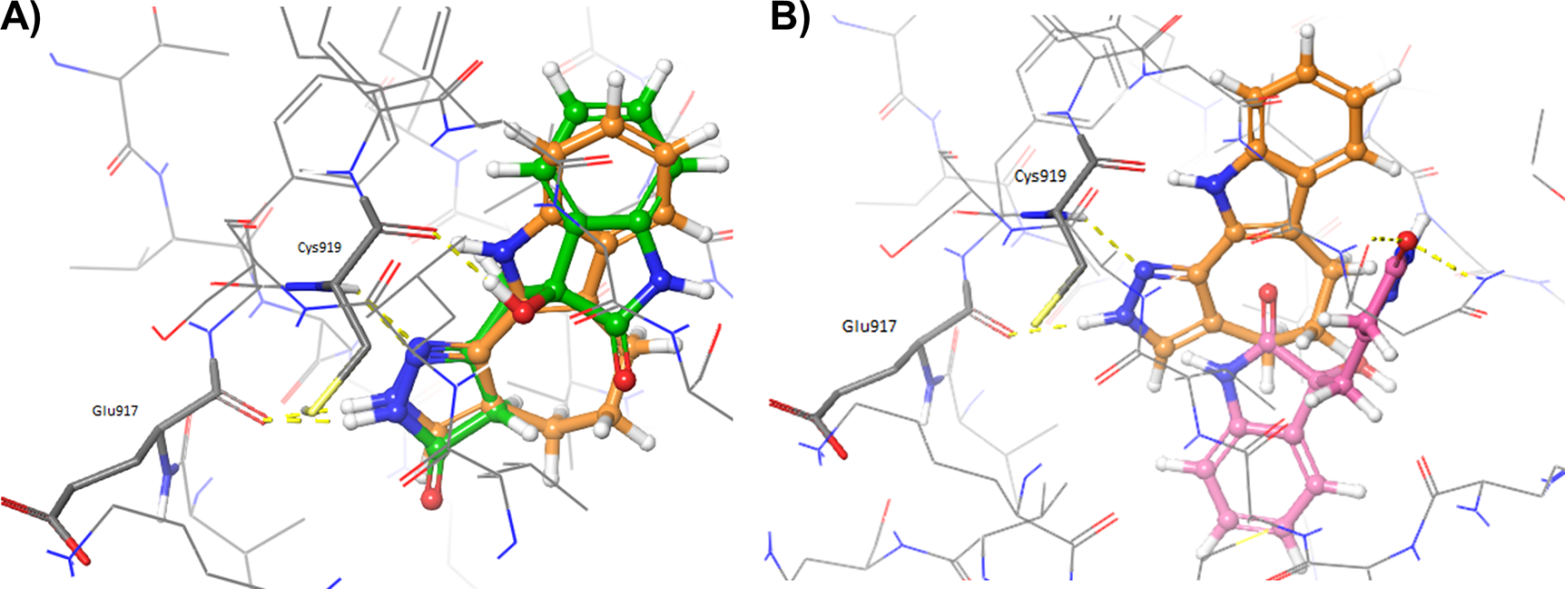
Crystallographic pose of the 4TT ligand (depicted as orange sticks) into the vascular endothelial growth factor receptor 2 (VEGFR-2) active site (Protein Data Bank code: 3VID), compared to the docked poses of A) oxindole_19 (depicted as green sticks) and B) oxindole_9 (depicted as pink sticks). Hydrogen bonds are depicted as yellow dashed lines.

Afterwards, the oxindole molecules were prepared with LigPrep (Schrödinger) and docked into the validated grid by using Glide, as described in the Methods section. ^31^ Results of the docking calculations performed with Glide for the oxindole_9 and oxindole_19 molecules are reported in Table 4. The complexes predicted by Glide for 4TT, oxindole_9 and oxindole_19 are made available in the PDB format as *Extended data.* ^36^ Figure 3 shows the comparison of the docked oxindoles and the 4TT ligand of VEGFR-2. In particular, the pyrazole ring of oxindole_19 perfectly overlapped with the corresponding moiety of 4TT, establishing H-bond interactions with the residues in the hinge region of the protein, as shown in Figure 3A. Indeed, the hydroxyl group of the oxindole_19 was predicted to establish one additional hydrogen bond with the backbone of Cys919, compared to the 4TT ligand. Moreover, a good overlap between the benzene ring of oxindole_19 and 4TT was also observed. On the contrary, oxindole_9 ( Figure 3B) was predicted to accommodate into the 3VID binding site in a conformation different to that experimentally observed for the 4TT crystallographic ligand. As a consequence, oxindole_9 was not predicted to form the essential H-bond interactions with the residues at the hinge region of the protein. Taken together, the results of these analyses showed that only oxindole_19 would be able to form favourable interactions with the residues lining the binding site of 3VID. To evaluate whether the different poses observed for the two oxindole ligands could derive by receptor flexibility, not taken into consideration in rigid docking performed with Glide, additional IFD calculations were performed as described in the Methods section. The adopted Induced Fit Docking protocol allowed to collect 20 minimized protein-ligand complexes for each oxindole molecule. Table 5 reports the poses with the best score for each of the investigated oxindole derivates.

**Table 4.** Results of the docking calculations of 4TT, oxindole_9 and oxindole_19 into the 3VID crystal structure.

Molecule	Glide Gscore (kcal/mol)
4TT	-8.2
oxindole_19	-8.0
oxindole_9	-6.0

**Table 5.** Results of the Induced Fit Docking calculations for 4TT, oxindole_19 and oxindole_9 into the 3VID crystal structure.

Molecule	Glide Gscore (kcal/mol)	IFD score (kcal/mol)
4TT	-9.5	-637.7
oxindole_19	-8.4	-640.0
oxindole_9	-8.6	-639.9

**Figure 4.**
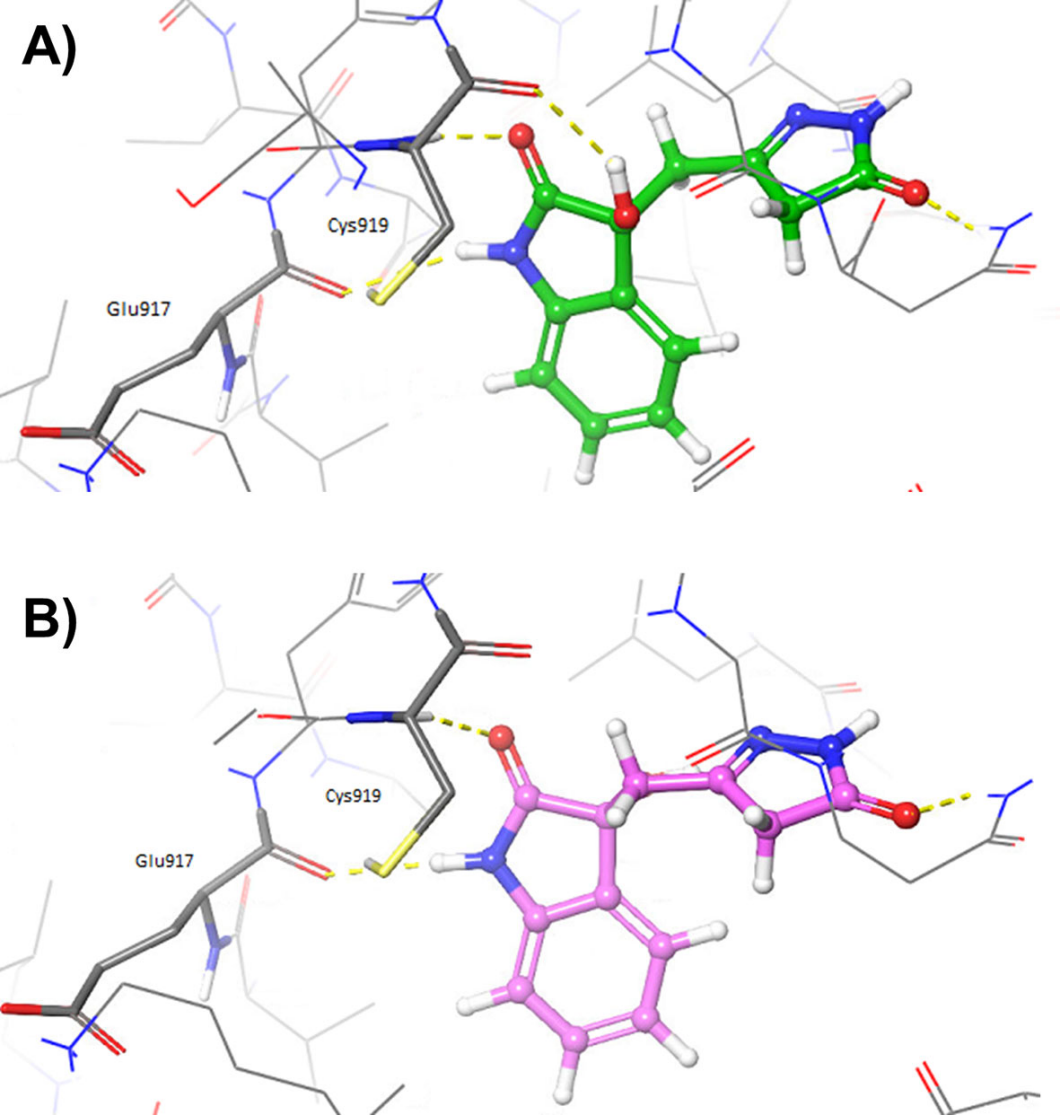
Induced Fit Docking poses obtained for A) oxindole_19 (depicted as green sticks) and B) oxindole_9 (depicted as pink sticks), into the vascular endothelial growth factor receptor 2 (VEGFR-2) active site (Protein Data Bank code: 3VID).

Interestingly, the pose previously predicted for oxindole_19 by rigid docking with Glide, in which the pyrazole ring interacts with hinge residues, was confirmed by IFD (see 3VID_oxindole_19_IFD_complex_bm1.pdb **,** *Extended data*). ^36^ Moreover, additional poses were also predicted with the IFD protocol for both oxindole_9 and oxindole_19, in which the oxindole ring forms hydrogen bonds with hinge residues Cys919 and Glu917 (see Figure 4 and *Extended data* files 3VID_oxindole_9_IFD_complex.pdb and 3VID_oxindole_19_IFD_complex_bm2.pdb). ^36^ Interestingly, these are the same types of interactions that have been previously observed for the VEGFR-2 inhibitor semaxinib, crystallized with the proto-oncogene tyrosine-protein kinase receptor Ret (PDB accession code: 2X2M). ^41^

### Experimental validation

Based on data collected from the ligand- and structure-based calculations described above, one of the identified targets was selected to provide a proof-of-concept on our repositioning hypotheses ( Table 2). To this end, we tested the enantiomeric pair consisting of oxindole_19 and oxindole_9 on isolated VEGFR-2 protein at Reaction Biology Corp, as described in the Methods section. These molecules showed significant shape similarity to already reported VEGFR-2 inhibitors, and good complementarity with the enzyme binding site according to docking analyses. However, the tested molecule turned out to be inactive on the target. This observation may be explained by taking into consideration the structure of known similar VEGFR-2 inhibitors. In particular, VEGFR-2 inhibitors as semaxinib are planar due to the presence of a double bonded methylene linker connecting the oxindole to the other aromatic ring (see Figure 1A). On the contrary, such a restrained linker is not present in the investigated molecules, resulting in higher flexibility (see Table 1). We therefore expect that introducing structural restraints into the investigated scaffolds would increase VEGFR-2 inhibitory activity. In the light of these results, docking alone was not able to correctly predict the activity of the ligand on VEGFR-2. We expect that more accurate scoring functions or the integration of docking with other structure-based approaches, such as molecular dynamics and binding-free energy calculations, similarly to what we previously discussed, may improve the computational predictions. ^6^ While oxindoles_19 and_9 did not show VEGFR-2 inhibitory activity, the performed *in silico* analyses allowed to identify further potential opportunities for drug repurposing that might be worth of future investigation with integrated *in silico* workflows and *in vitro* studies ( Table 2).

## Conclusions

Several studies have demonstrated that drug repurposing could represent a valuable alternative strategy to traditional *de novo* drug discovery. Indeed, drug repositioning presents many advantages over traditional drug development as it allows, for example, to efficiently exploit known information for already investigated molecules, including data available from safety and formulation assessments. Nevertheless, drug repurposing strategies usually require less time, expenses and risks of failure compared to *de novo* drug discovery. As originally conceived, the aim of drug repurposing is to find new therapeutic indications for already existing drugs, albeit such an approach has already been demonstrated to provide novel valuable opportunities for natural products and already synthesized molecules as well. Based on these premises, in this work we performed extensive 2D and 3D similarity calculations on the tautomeric state potentially accessible at physiological pH by two enantiomeric molecules bearing an oxindole scaffold. The compounds were identified within an effort to synthesize hydroxy-pyrazole and 3-hydroxy-oxindole chemical moieties starting from isatins, ^18^ and could hold great premises for the development of leads to be investigated, for example, in pharmacological and agrochemical contexts. Interestingly, an analysis of the results from the performed ligand similarity estimations highlighted potential activity for some of the oxindole derivates towards a set of therapeutically relevant targets ( Table 2). One of the identified targets, *i.e.*, VEGFR-2, was also selected to be further investigated by structure-based analyses, followed by experimental validation on one oxindole candidate. In particular, the structure-based analyses performed on VEGFR-2 highlighted good steric and electronic complementarity between oxindole_19 and oxindole_9, and the binding site residues of this target. Based on these results, the racemic mixture of the investigated oxindole compounds was experimentally tested *in vitro* on VEGFR-2. While the compounds did not result to be active on VEGFR-2, a number of additional targets emerged from the ligand-based analyses, which might be worth considering for further *in silico* and *in vitro* experimentation in future research programs. To the best of our knowledge, this is the first study based on an *in silico* drug repurposing approach performed on ligands bearing an oxindole scaffold, enantiomeric pairs and a complete tautomeric description. Considering the relevance of this type of scaffold in different research areas, novel valuable therapeutic opportunities could be envisioned on other targets for such oxindole derivates.

## Data availability

### Underlying data

Ligands and activity data employed in the similarity-based analyses were downloaded from DrugBank, Protein Data Bank and ChEMBL databases (accessed on October 13 ^th^, 2020). A complete list of accession numbers as mentioned in the text, and their corresponding databases sources is provided as extended data (list_accession_codes.csv).

### Extended data

Zenodo: Extended data for Manuscript: Identification of potential biological targets of oxindole scaffolds via *in silico* repositioning strategies, https://doi.org/10.5281/zenodo.6038245. ^36^

This project contains the following extended data:

• list_accession_codes.csv (a complete list of accession numbers as mentioned in the text, and their corresponding database sources).
• 2D_similarity_data_oxindoles_9-19.csv (results of 2D fingerprint-based similarity analyses for oxindole_9 and oxindole_19. Results were filtered according to the following thresholds: Tc _MACCS_ ≥ 0.7, Tc _ECFP4_ ≥ 0.3, Tc _ECFP6_ ≥ 0.4, Tc _FCFP4_ ≥ 0.3 and Tc _FCFP6_ ≥ 0.4)
• 3VID_4TT_GlideSP_complex.pdb (ligand-protein complexes predicted by rigid docking calculations for crystallographic ligand 4TT)
• 3VID_oxindole_9_GlideSP_complex.pdb (ligand-protein complexes predicted by rigid docking calculations for oxindole_9)
• 3VID_oxindole_19_GlideSP_complex_bm1.pdb (ligand-protein complexes predicted by rigid docking calculations for oxindole_19)
• 3VID_oxindole_9_IFD_complex.pdb (best scoring ligand-protein complexes predicted by Induced Fit Docking for oxindole_9)
• 3VID_oxindole_19_IFD_complex_bm1.pdb (ligand-protein complexes predicted by Induced Fit Docking for oxindole_19)
• 3VID_oxindole_19_IFD_complex_bm2.pdb (best scoring ligand-protein complexes predicted by Induced Fit Docking for oxindole_19)

All data are available under the terms of the Creative Commons Attribution 4.0 International license (CC-BY 4.0).
