# Supplementary material for: Identification of potential biological targets of oxindole scaffolds via in silico repositioning strategies
Source: F1000Res. 2022 Mar 23;11:Chem Inf Sci-217. Originally published 2022 Feb 23. [Version 2] doi: 10.12688/f1000research.109017.2 (PMC10521104; doi:10.12688/f1000research.109017.2)
Supplement: Peer review history

# Reviewer report: Reviewer response for version 2

**DOI:** 10.5256/f1000research.122404.r128555

**Published:** 2022-03-23

**Authors:** Giuseppe Felice Mangiatordi

**Affiliations:** 1 CNR—Institute of Crystallography, Bari, Italy

I do not have any comment to add.

Is the work clearly and accurately presented and does it cite the current literature?

Yes

If applicable, is the statistical analysis and its interpretation appropriate?

Yes

Are all the source data underlying the results available to ensure full reproducibility?

Yes

Is the study design appropriate and is the work technically sound?

Yes

Are the conclusions drawn adequately supported by the results?

Yes

Are sufficient details of methods and analysis provided to allow replication by others?

Yes

Reviewer Expertise:

Ligand- and structure-based drug design

I confirm that I have read this submission and believe that I have an appropriate level of expertise to confirm that it is of an acceptable scientific standard.

# Reviewer report: Reviewer response for version 1

**DOI:** 10.5256/f1000research.120468.r125431

**Published:** 2022-03-03

**Authors:** Giuseppe Felice Mangiatordi

**Affiliations:** 1 CNR—Institute of Crystallography, Bari, Italy

This paper by Tinivella *et al.* reports an *in-silico* repositioning strategy aimed at selecting new pharmaceutical targets for a library of already synthesized oxindole-based compounds. The topic is of interest, the manuscript is written clearly and convincingly most of the time, and can be reasonably considered an interesting contribution for the F1000Research audience. The reviewer also appreciated that the authors reported the obtained (disappointing) experimental data. The applied methodology is technically sound and the presented data reproducible.

**Minor points**

- Reference 6 seems to be not appropriate as it is not related to the current COVID-19 pandemic. I suggest moving it to the first part of the introduction.  
- *In-silico* drug repurposing strategies have been undertaken by many groups in the last two years in the attempt to quickly fight COVID-19. Examples are given by:
  - https://doi.org/10.1186/s12859-021-03988-x
  - https://doi.org/10.3390/molecules25173830
  - https://doi.org/10.3389/fchem.2020.594009

          The introduction would benefit from more references on this topic to properly emphasize the importance of this computational approach.

- Density-functional theory (DFT) data: The used DFT functional should be reported in the Tautomeric Stability section.

Is the work clearly and accurately presented and does it cite the current literature?

Yes

If applicable, is the statistical analysis and its interpretation appropriate?

Yes

Are all the source data underlying the results available to ensure full reproducibility?

Yes

Is the study design appropriate and is the work technically sound?

Yes

Are the conclusions drawn adequately supported by the results?

Yes

Are sufficient details of methods and analysis provided to allow replication by others?

Yes

Reviewer Expertise:

Ligand- and structure-based drug design

I confirm that I have read this submission and believe that I have an appropriate level of expertise to confirm that it is of an acceptable scientific standard.

## References

[1] : In silico drug repositioning based on the integration of chemical, genomic and pharmacological spaces. *BMC Bioinformatics* .2021;22(1) : 10.1186/s12859-021-03988-x 52 10.1186/s12859-021-03988-x 33557749PMC7868667

[2] : Drug Repurposing for Candidate SARS-CoV-2 Main Protease Inhibitors by a Novel In Silico Method. *Molecules* .2020;25(17) : 10.3390/molecules25173830 10.3390/molecules25173830 32842509PMC7503980

[3] : Repurposing Known Drugs as Covalent and Non-covalent Inhibitors of the SARS-CoV-2 Papain-Like Protease. *Front Chem* .2020;8: 10.3389/fchem.2020.594009 594009 10.3389/fchem.2020.594009 33304884PMC7701290

# Response

**Published:** 2022-03-12

**Authors:** Giulio Rastelli

**Affiliations:** University of Modena and Reggio Emilia, Italy

We would like to thank the reviewer for the valuable suggestions. We have revised the introduction section of the manuscript to include a more thorough description on the importance of computational approaches in drug repurposing. Moreover, we have also revised the bibliographic references accordingly, as suggested. In addition, we have also provided additional experimental details on the approach adopted for the evaluation of the tautomeric preference calculations.

# Reviewer report: Reviewer response for version 1

**DOI:** 10.5256/f1000research.120468.r125433

**Published:** 2022-03-01

**Authors:** Carmine Varricchio

**Affiliations:** 1 School of Pharmacy and Pharmaceutical Sciences, Cardiff University, Cardiff, UK

In this study, Tinivella *et al.* describe an *in silico *drug repositioning strategy using known bioactive oxindole-based compounds **.** The *in silico* workflow is well presented, and the authors provide a detailed step-by-step procedure for identifying potential repurposing drugs. Although the biological results showed a lack of activity for the *in silico *identified compounds, the manuscript provides valuable guidelines for future repositioning strategies. However, the authors might consider expanding the discussion section on the biological activity, adding a potential explanation for the lack of activity and/or potential improvement in their *in silico* workflow.

Is the work clearly and accurately presented and does it cite the current literature?

Yes

If applicable, is the statistical analysis and its interpretation appropriate?

Not applicable

Are all the source data underlying the results available to ensure full reproducibility?

Yes

Is the study design appropriate and is the work technically sound?

Yes

Are the conclusions drawn adequately supported by the results?

Partly

Are sufficient details of methods and analysis provided to allow replication by others?

Yes

Reviewer Expertise:

Drug discovery, computational and medicinal chemistry

I confirm that I have read this submission and believe that I have an appropriate level of expertise to confirm that it is of an acceptable scientific standard.

# Response

**Published:** 2022-03-12

**Authors:** Giulio Rastelli

**Affiliations:** University of Modena and Reggio Emilia, Italy

We would like to thank the reviewer for the comments. 

 In the revised version of the manuscript, we provided a potential explanation for the observed lack of activity for oxindole_9 and oxindole_19 compounds towards VEGFR-2. The compounds resulted similar from a shape-based point of view with respect to known inhibitors of this target and showed good target complementary according to docking. However, the investigated compounds present some structural differences with respected to known VEGFR-2 inhibitors. In particular, we discussed the higher flexibility of the methylene linker of the investigated oxindole scaffold, which could potentially hamper biological activity.

 We propose that the integration of docking with molecular dynamics and binding free energy calculations could improve the prediction performance of the adopted *in silico *workflow, thus resulting in more accurate predictions.
